# Maternal exposure to work schedule unpredictability and child behavior

**DOI:** 10.1111/jomf.12800

**Published:** 2021-09-21

**Authors:** Daniel Schneider, Kristen Harknett

**Affiliations:** ^1^ Harvard Kennedy School and Department of Sociology Harvard University Cambridge Massachusetts USA; ^2^ Department of Social and Behavioral Sciences University of California, San Francisco San Francisco California USA

**Keywords:** child behavior, inequality, precarious work, work scheduling

## Abstract

**Objective:**

This article estimates the association between maternal exposure to unpredictable work schedules in the service sector and child internalizing and externalizing behavior.

**Background:**

Precarious work is widespread and characterized by low wages, few benefits, and nonstandard schedules. But working parents, especially in the service sector, contend with unpredictable work schedules as well. These schedules have negative consequences for workers, but may also perpetuate inequality across generations by negatively affecting children.

**Method:**

This article takes advantage of novel survey data from The Shift Project, covering 2,613 mothers (surveyed 2017–2019) working in the service sector with children (mean child age of 7.5), to examine the association between maternal work schedules and child behavior as well as the mediators of this relationship.

**Results:**

Maternal exposure to unpredictable work schedules is associated with children's externalizing and internalizing behavior. Mediation analysis shows that for parents with the most unpredictable schedules, this aspect of job quality operates on children's behavior by increasing household economic insecurity, reducing developmental parenting time, and diminishing maternal well‐being.

**Conclusion:**

These results demonstrate that work scheduling conditions may have consequences not just for workers themselves but also for their children.

**List of Abbreviations/Acronyms**
ACS: American Community Survey
CBCL: Child Behavior Check‐list
CPS: Current Population Survey
FSM: Family Stress Model
GSS: General Social Survey
KHB: Karlson‐Holm‐Breen
NLS: National Longitudinal Survey
NLSY79: National Longitudinal Survey of Youth ‐ 1979 Cohort
PSID: Panel Study of Income Dynamics
SES: Socioeconomic Status

## INTRODUCTION

Environmental and contextual forces exert a powerful influence on child health and well‐being (Bronfenbrenner, 1979). Of particular importance, parental employment affects children by shaping family processes and household economic security. However, since the 1970s, employment in the United States has become much more precarious (Kalleberg, 2009), especially among workers at the bottom of the income and occupational distribution (Fligstein & Shin, 2004). Precarious work is characterized by low wages, few benefits, and nonstandard work hours that encompass evenings, nights, and weekends (Kalleberg, 2011; Presser, 1999). More recently, scholars and policymakers have identified a new form of employment precarity: unpredictable work schedules. Many workers receive their work schedules only a few days in advance, scheduled work hours and work days may change substantially week‐to‐week, and shifts may be changed, canceled, or added at the last minute (Golden, 2001; Lambert, 2008).

Unpredictable work schedules (often also referred to as precarious schedules, “just in time” schedules, unstable schedules, or irregular schedules) are widespread in the retail and food service sectors (Golden, 2001; Henly et al., 2014). These sectors are of particular importance in part because of their size—in the United States, almost 20% of workers are employed in retail or food service and 1 in 10 children aged 5–18 years have a parent who works a service job in these sectors (author's calculations from 2015 american community survey [ACS]). The retail and food service sectors also represent the single largest concentration of low‐wage workers (Osterman & Schulman, 2011), and thus, parental exposure to precarious job conditions in this sector has the potential to magnify inequality across generations. Finally, the COVID‐19 pandemic has placed a spotlight on the difficult conditions facing essential workers in the service sector.

Since many workers exposed to unpredictable schedules are parents with low incomes, unpredictable work schedules have important implications for the inequality of child behavior. Unpredictable scheduling practices may affect children through several pathways. Children whose parents are exposed to such scheduling practices may experience higher levels of household economic strain (Schneider & Harknett, 2020), more time‐based conflict that could deplete quality time with parents (Greenhaus & Beutell, 1985), and may experience more parental stress (Schneider & Harknett, 2019a).

While there is evidence that other dimensions of precarious work—such as stable, but nonstandard hours—negatively affect child well‐being (i.e., Li et al., 2014), the evidence based on the effects of unpredictable scheduling practices on child behavior is very limited. Data sources containing information on both parental work schedules and child outcomes are rare, and workers in low‐wage unstable jobs are difficult to sample. We draw on new data collected by The Shift Project from 2,613 mothers of children aged 2–15 years working in the retail and food service sectors. We focus on mothers given that women continue to do a disproportionate share of care work in the contemporary United States (Yavorsky et al., 2015). Shift Project data contain detailed measures of maternal exposure to unpredictable scheduling, of mediating variables such as household financial insecurity and maternal stress and well‐being, and child behavior outcomes.

We find that there is a high degree of exposure to unpredictable work scheduling practices among mothers in the retail and food service sectors. We then show that children whose mothers are exposed to such practices, specifically on‐call shifts, last‐minute schedule changes, or short advance notice of work schedules, exhibit more internalizing and externalizing behavior. We show that these associations are significantly mediated by household economic insecurity, developmental parenting time, and maternal well‐being, with the latter playing the largest explanatory role.

## BACKGROUND

Children's growth and development is shaped by their ecological contexts—the complex and multilevel set of systems that define a child's environment (Bronfenbrenner, 1979, 1986). These early life conditions matter a great deal for setting life‐long trajectories of achievement and attainment (i.e., Heckman, 2006). Children's environments are a product of the neighborhoods they live in, the schools they attend, and also, crucially, the families in which they grow up (Duncan & Murnane, 2011; McLanahan, 2004). Children's relationships with their parents, including time spent together and positive interactions, are an important determinant of child well‐being (Bronfenbrenner, 2005), and family contexts appear to shape disparities in child achievement (Larson et al., 2015; Waldfogel & Washbrook, 2011). However, these familial contexts are importantly shaped by broader economic and structural forces. The jobs that parents hold exert a strong influence on family life and the experiences of their children (Edwards & Rothbard, 2000; Menaghan, 1991; Shonkoff & Phillips, 2000). While job quality is often defined in terms of wages and benefits (Kalleberg, 2009), work schedules also are an important dimension of job quality that affect parent and child well‐being (Presser, 2003). While prior work on scheduling has focused on nonstandard shifts (Li et al., 2014) or on schedule flexibility and control (Kelly et al., 2011), the reality of work scheduling for parents in the large retail and food service sector is of unpredictable work schedules (Lambert et al., 2014; Schneider & Harknett, 2019a).

Unpredictable schedules in particular may spill over to affect children's behavior through several pathways. Children whose parents are exposed to such unpredictable scheduling practices may experience higher levels of material deprivation, which is likely to negatively affect well‐being (Duncan & Brooks‐Gunn, 1997) as well as higher levels of income instability, which may directly affect cognitive processes and development (Gennetian & Shafir, 2015; Mani et al., 2013). Parents who have unpredictable work schedules may also experience time‐based conflicts (Greenhaus & Beutell, 1985) that affect parental investments in child development by depleting parental time with children (Conger & Donnellan, 2007). Parental exposure to unpredictable scheduling practices may increase parental stress and depression and so diminish the quality of parent–child interactions (Conger & Elder, 1994; McLoyd, 1990; Yeung et al., 2002). Each of these mechanisms may be particularly important for working mothers, given that mothers tend to have primary responsibility in caring for dependent children (Bianchi & Milkie, 2010).

## MECHANISMS

Mothers' unpredictable work schedules are likely to affect child behavior through the specific mechanisms of increased household financial insecurity, reduced developmental parenting time, and diminished maternal well‐being.

### Household economic insecurity

First, mothers' exposure to unpredictable scheduling practices at work may serve to negatively affect child well‐being by increasing economic volatility and household material hardship. Variable hours may, mechanically, lead to income volatility for hourly workers, especially if that variability makes it difficult for workers to hold secondary jobs that might otherwise be used to smooth earnings. Recent survey data show that low‐ and moderate‐income workers name an irregular work schedule as the most common cause of intra‐year income volatility (Federal Reserve Board, 2014). In a financial diary study, a sudden drop in weekly work hours was one of the main reasons families experienced negative income shocks (Morduch & Schneider, 2014). Last‐minute changes to work schedules through short‐notice scheduling or on‐call shifts may also make it difficult for workers to actually make the shifts that they are scheduled for, increasing income volatility but also household material hardship (Edin & Shaefer, 2015; Golden, 2015; Haley‐Lock, 2011; Luce et al., 2014; Zeytinoglu et al., 2004). Recent research focusing specifically on retail and food service workers finds that exposure to unpredictable work schedules, in the form of on‐call shifts, canceled shifts, short advance notice, last‐minute changes to schedule timing, and work hour volatility, increases the risk of material hardships, net of controls for hourly wage, and household income (Schneider & Harknett, 2020).

Economic deprivation and economic insecurity appear, in turn, to have direct effects on child behavior. Children may observe family economic stress and be affected by it, even absent changes to parenting behaviors (Leininger & Kalil, 2014). Furthermore, hardships could result in relegation to substandard housing, which could result in exposure to damaging levels of lead and other pollutants that can seriously negatively affect child behavior (Brooks‐Gunn & Duncan, 1997; Duncan et al., 2014; Evans, 2004). Low and volatile parental incomes can lead to food insecurity and poor nutrition (Bhattacharya et al., 2004), which in turn negatively affect child both internalizing and externalizing behavior (Slopen et al., 2010; Whitaker et al., 2006). Taken together, this previous literature suggests that mothers' exposure to unpredictable schedules is likely to lead to economic insecurity, with negative consequences for child behavior.

### Developmental parenting time

Unpredictable work schedules also appear to interfere with mothers' ability to establish regular routines for their children, to make time for regular engagement in developmental activities such as reading and homework help, and to facilitate children's involvement in organized enrichment activities. Workers who have less advance notice of their schedules and less schedule control report less ability to do things such as schedule a doctor's appointment or cook a meal at home (Henly & Lambert, 2014). Furthermore, mothers' exposure to irregular work schedules reduces shared family meals (Han & Waldfogel, 2007; Hsueh & Yoshikawa, 2007) and low‐wage mothers' lack of flexibility and control over their schedules substantially interferes with parental engagement with children's schools and school‐based activities (Haley‐Lock & Posey‐Maddox, 2016). While time‐based conflicts may often be thought of in the context of insufficient time (Schulte, 2014), in the case of unpredictable schedules it is the volatility and uncertainty of time that is expected to upset family rhythms and interfere with parenting (Gerstel & Clawson, 2018).

These forms of parental investment in turn affect child behavior. Mothers' parenting behaviors shape children's internalizing and externalizing behavior (Galambos et al., 2003; Reitz et al., 2006). Increased maternal time in activities with children is negatively related to internalizing and externalizing behavior (Estes, 2004).

### Maternal well‐being

Mothers who do not know how many hours they will work, when their shifts will occur, or if they might be sent home early or asked to stay late are likely to experience elevated stress and depression. While limited, existing research on the effects of unpredictable schedules on worker well‐being suggests that these scheduling practices do increase stress. Workers exposed to unpredictable hours report higher levels of stress and irritability (Zeytinoglu et al., 2004) as well as more depressive symptoms and less happiness (Schneider & Harknett, 2019a). Retail workers with unpredictable work schedules also report lower quality sleep (Harknett et al., 2020; Lambert et al., 2019; Schneider & Harknett, 2019a). Mothers who work an irregular schedule during the first year of their infants' lives reported more depressive symptoms (Grzywacz et al., 2016) and workers exposed to unpredictable scheduling practices had more anxiety and feelings of loss of personal control (Henly & Lambert, 2014). Importantly, these effects of precarious schedules are likely to be net of effects on household economic insecurity (Schneider & Harknett, 2019a).

A large body of theoretical and empirical research in turn makes the case that parental stress and depression negatively affect child behavior. This work generally proceeds from the Family Stress Model (FSM) (Conger & Elder, 1994; McLoyd, 1990). The FSM posits that financial problems and strain negatively affect child well‐being by increasing parental strain and depression, which in turn results in harsh, inconsistent, or uninvolved parenting (Conger et al., 2010). Empirical research has shown that lower economic well‐being negatively affects teachers' ratings of social behavior by reducing parental psychological well‐being (Mistry et al., 2002) and that low family incomes increase child behavioral problems by increasing maternal emotional distress (Yeung et al., 2002).

## PRIOR EMPIRICAL RESEARCH

Scholars have long been concerned with the effects of parental work time on parent and child well‐being (i.e., Bianchi, 2000; Brooks‐Gunn, Han, & Waldfogel, 2010; Han et al., 2001). Prior research has also now carefully documented the effects of nonstandard work schedules on child well‐being (see reviews by Dunifon et al., 2012 and Li et al., 2014). Parental exposure to nonstandard schedules is associated with both internalizing and externalizing behavior (Joshi & Bogen, 2007; Li et al., 2014), though there is also evidence that externalizing may be most affected by nonstandard work hours (Hsueh & Yoshikawa, 2007). Separate research in sociology and epidemiology, primarily focusing on professionals, has shown how limited flexibility in the workplace depresses worker well‐being (Glass & Estes, 1997) and parental time with children (Davis et al., 2015).

While both nonstandard schedules and inflexible schedules are important dimensions of job quality, they are quite distinct from the unpredictable schedules that appear common in the retail sector and that are the object of recent policy attention. Yet, there is little research that directly examines the effects of unpredictable schedules on child behavior. However, as reviewed above, early research on unpredictable scheduling does provide an important basis for the hypothesis that unpredictable schedules might affect child behavior by showing empirical evidence of how unpredictable schedules are related to household economic insecurity, time‐based conflict, and stress‐based conflict—the factors hypothesized to mediate relationships between mothers' work schedules and their children's behavior.

While few studies directly examine the relationship between mothers' unpredictable schedules and child behavior, there are important exceptions. Maternal variable schedules are associated with more problem behavior and lower social competence for infants (Grzywacz et al., 2016). Maternal work schedules that are both variable and nonstandard are associated with lower teacher‐reported school engagement and performance and higher externalizing behaviors (Hsueh & Yoshikawa, 2007). Furthermore, week‐to‐week fluctuations in maternal work hours are also negatively associated with child behavior problems including internalizing and externalizing behavior (Johnson et al., 2012). However, one study using Fragile Families data, Dunifon et al. (2013), finds no association between maternal work at “different times each week” and children's aggressive or anxious behaviors when compared against mothers who did not work, and actually finds that working “different times each week” is associated with fewer behavior problems than working a night shift.

## LIMITS OF EXISTING RESEARCH

Further research on the relationship between unpredictable work schedules and child well‐being is constrained by a lack of current data that contain detailed measures of scheduling, child well‐being, and mediating mechanisms. Both Johnson et al. (2012) and Hsueh and Yoshikawa (2007) rely on state‐specific data that is now 20 years old. The other extant studies rely on either the Fragile Families and Child Wellbeing study or the National Longitudinal Survey of Youth‐Child Supplement. Both contain useful measures of child well‐being and some measures of parental work schedules. However, the measurement of schedules is very limited and is much better suited to capturing work during nonstandard hours than unpredictable schedules as experienced through practices such as on‐call work, canceled shifts, and changes in shift timing. Furthermore, the available measure of variable work cannot separate the distinct experiences of high‐socioeconomic status (SES) workers who have the autonomy and control to achieve desirable flexibility from the low‐SES workers who have little control and are subject to employer‐driven instability (Han et al., 2010; Li et al., 2014).

Other large‐scale datasets are even more limited. Most existing surveys do not measure schedule instability (panel study of income dynamics [PSID], ACS, current population survey [CPS], national longitudinal survey of youth – 1979 cohort [NLSY79]). Those surveys that carefully measure scheduling practices lack data on child outcomes (EINet Polls). In addition, the few datasets that both measure scheduling and come close to outcomes of interest (NLSY97 for 2014 and general social survey [GSS] in 2016) have insufficient power to study the policy‐relevant sample of mothers who are managing the dual responsibilities of low‐wage work and caring for dependent children.

## METHOD

The data for this study were collected as part of The Shift Project, an ongoing study of low‐wage work that began in 2016 using an innovative data collection approach. The Shift Project takes the advantage of sophisticated targeting capabilities available through Facebook's advertising platform to recruit workers to complete an online survey. The Facebook advertising platform allows advertisers to select subpopulations of users to see their advertisements. To construct The Shift Project data, survey recruitment advertisements were targeted to active users on Facebook and Instagram who resided in the United States, were over the age of 18 and under the age of 65, and were employed by one of 131 large retail or food service companies.

Advertisements are placed in the Facebook desktop or mobile newsfeeds or Instagram account of targeted users with an invitation to participate in a short survey. The advertisement included a picture of a worker in a uniform and a setting designed to be similar to the worker's workplace, and the advertisement text named the employer in the survey invitation for added resonance. The survey is a repeated cross‐sectional design, with a rotating subset of the 131 employers targeted at each wave. The survey was fielded using this method at six time points: (1) April–June 2017, (2) September–November 2017, (3) February–June 2018, (4) October–November 2018, (5) February–April 2019, and (6) September–November 2019. In all, across the 131 employers and six repeated cross sections, the respondents who compose the analysis sample here were recruited via 349 unique advertisements.

Users who clicked on the link in the advertisement were redirected to an online survey hosted on Qualtrics, which contained modules on work schedules, demographics, economic security, health, parenting, and, for parents, child well‐being. The front page of the survey contained introductory information and a consent form that respondents could assent to by clicking and then advance to the survey instrument. Respondents who completed the survey and provided contact information were entered into a drawing for an iPad. Approximately 1.2% of advertisement displays yielded survey data. Although these response rates are lower than obtained in many probability‐sample phone surveys, a large sample of working parents employed in the service sector would be difficult if not impossible to reach through traditional methods given the absence of an appropriate sampling frame. A more detailed description of The Shift Project data collection procedures is described in Schneider and Harknett (2019b).

The Shift Project method of collecting data results in a strategically targeted, non‐probability sample, raising concerns about representativeness and bias (Groves, 2011; Smith, 2013). Although the use of Facebook as a sampling frame will largely exclude workers without Internet access and who are not active on Facebook or Instagram, recent estimates suggest that 84% of working adults aged 18 to 50 years are active on Facebook or Instagram (Greenwood et al., 2016) and this share does not vary by household income (Author's tabulations from Pew, 2018). In addition, with a similar data collection approach using Facebook, Zhang et al. (2017) compare respondents drawn from Facebook and the American Community Survey in terms of veteran status, homeownership, and nativity and find a high degree of similarity. For The Shift Project data specifically, prior validation work shows that univariate distributions of work scheduling exposures and bivariate associations are similar in the Shift data as in the CPS and NLSY97 (Schneider & Harknett, 2019b).

Using this method, The Shift Project recruited 2,613 mothers with a focal child age 2 or older and less than age 16 to complete the survey. Of these respondents, 8.6% had some item nonresponse on the analysis variables (described below). We use the Amelia II package in R to multiply impute the missing data, constructing 10 implicates that we use for descriptive statistics and analyses. We additionally post‐stratify and weight The Shift Project mothers subsample along the dimensions of race/ethnicity, age, gender, and education to the “gold‐standard” of parents in the same set of occupations and industries who are captured in the 2008–2017 American Community Survey. We additionally weigh the data to ensure that the representation of mothers by the employer matches the relative employee sizes of each employer in the data. We do so by calculating total employment at each of the 131 firms in the data from the Reference USA database, collapsing establishment‐level employment from 365,294 establishments into firm‐level counts. We apply these weights to all of the analyses.

## KEY VARIABLES

### Child behavior

First, mothers complete the Child Behavior Check‐List Brief Problem Monitor (CBCL), which contains items that we use to construct scales for internalizing and externalizing behavior (Achenbach et al., 2011). The survey collects a full roster of household children's ages and genders. For mothers with just one child, the survey asks the Brief Problem Monitor with regard to that child. For mothers who report more than one child on the roster, the survey selects a focal child who is closest to age 7. This yields a distribution of focal child ages, centered on age 7, which range from age 2 to age 15. Approximately 25% of the sample aged 2‐4 years, the next quartile aged 5–7 years, the third quartile aged 8–10 years, and the top quartile aged 11–15. The age range of focal children in our sample includes only children at ages for which the CBCL was designed and has been shown to return reliable assessments (Achenbach & Ruffle, 2000; Mazefsky et al., 2011; Naar‐King et al., 2014).

For internalizing, the scale (α = .84) is based on mothers' reports on a 0–2 scale of whether it is “not true,” “sometimes true,” or “very true” that the focal child (1) feels worthless or inferior; (2) is too fearful or anxious; (3) feels too guilty; (4) is self‐conscious or easily embarrassed; (5) is unhappy, sad, or depressed; and (6) worries. For externalizing, the scale (α = .84) is based on mothers' reports using the same response options to items on whether the focal child (1) argues a lot; (2) destroys things belonging to his/her family or others; (3) is disobedient at home; (4) is disobedient at school/care; (5) is stubborn, sullen, or irritable; (6) has temper tantrums or hot temper; and (7) threatens people. These alphas meet or exceed the benchmarks reported by Piper et al. (2014) in their validation study. All of these items are introduced with text that instructs mothers to, “please rate each item to describe (CHILD INITIALS) now or within the past month.” The reports are summed across items for each scale. The internalizing scale has a mean of 1.7 and the externalizing scale a mean of 3.6. These scores are higher than the means of 1.5 and 2.5 reported by Piper et al. (2014) in their validation study using a convenience sample of Oregon caregivers.

### Schedule unpredictability

We measure the key independent variable, schedule unpredictability, with several indicators. First, we measure exposure to “on‐call” shifts. Mothers are coded “1” if they respond affirmatively to the question, “In the past month or so, have you ever been asked to be ‘on‐call’ for work at (EMPLOYER NAME)? By ‘on‐call,’ we mean you have to be available to work, and you find out if you are needed to work just a few hours before your shift,” and coded “0” otherwise. Second, we measure exposure to last‐minute changes to shifts. Mothers are coded “1” if they respond affirmatively to the question, “In the past month or so, did your employer ever change the timing or the length of your scheduled shift at (EMPLOYER NAME)? For example, your employer asked you to come in early or late, or asked you to leave early or to stay later than the hours you were originally scheduled for,” and coded “0” otherwise. Third, we measure the amount of advanced notice of the work schedule that mothers usually receive (“How far in advance do you usually know what days and hours you will need to work at (EMPLOYER NAME)?”), and we distinguish 0–2 days of advance notice, 3–6 days, 1–2 weeks, and 2 weeks or more advance notice. These items effectively capture the extensive margin of schedule instability and unpredictability, but they do not capture the intensive margin—for instance the number of on‐call shifts or the number of timing changes. This is a limitation of the measurement.

We use these items individually and to create an additive scale summarizing maternal schedule unpredictability (range 0–3). To do so, we construct a dichotomous measure of advance notice coded as “0” for those with at least 1 weeks' advance notice and “1” for those who receive less than 1 weeks' notice, and we sum this measure with the dichotomous measures of timing change and on‐call shifts.

### Mediating factors

We measure three types of mediators: household economic insecurity, developmental parenting time, and maternal well‐being. For each, we construct scale variables that combine multiple indicators. This approach allows us to decompose the contribution of these constructs to mediating the relationship between unpredictable scheduling and child behavior.

First, we measure household economic insecurity to capture the pathway in which mothers' unpredictable work schedules negatively affect children's well‐being by increasing deprivation and economic volatility in the household. We construct a scale variable (α = .6) that is composed of three items. For the first item, financial fragility, mothers report their capacity to cope with a $400 expense shock in the next month with response options of “I am certain I could come up with the full $400,” “I could probably…,” “I could probably not…,” and “I am certain that I could not come up with $400” (Federal Reserve, 2014; Lusardi, Schneider, & Tufano, 2011). The second item, income volatility, is coded as “1” if mothers report that their household income “goes up and down” from week to week versus “is basically the same” (Federal Reserve, 2018). For the third item, mothers report on in a typical month, “how difficult is it for you to cover your expenses and pay all your bills?” with responses of “very difficult,” “somewhat difficult,” and “not at all difficult.”

Second, we measure developmental parenting time in order to capture the pathway by which maternal unpredictable work schedules might negatively affect children's behavior by upsetting mothers' ability to spend time with their children. We construct a scale variable (α = .80) that is composed of four items. Each item gauges the frequency (never in the past month, one to two times in the past month, once a week, several times a week, every day) with which mothers report spending time with their children (1) “working on homework or reading a book together,” (2) “participating in indoor activities together (such as arts and crafts or board games),” (3) “having a meal together,” and (4) “participating in outdoor activities together (like going for a walk or to a playground).”

Third, we measure maternal well‐being to capture the pathway by which maternal unpredictable work schedules negatively affect children's behavior by increasing mothers' stress and depressing their mood such that mother–child relationships are strained and interactions are of lower quality. We construct a scale variable (α = .61) composed of three sets of indicators. The first is coded as “1” if mothers report being “pretty happy” or “very happy” as opposed to “not too happy these days.” The second is coded as “1” if mothers report their sleep quality as “very good” or “good” as opposed to “fair” or “poor” over the past month. The third item is coded as “1” if mothers score 13 or higher on the Kessler‐6 scale of psychological distress with reference to affect over the past month, the generally accepted cutoff for significant psychological distress (Lee et al., 2012).

All of these mediating variables are designed to capture reports in reference to either the present or the prior month. This ensures that the reference period for the mediators is contemporaneous with the reference period for the measures of both maternal unpredictable scheduling and child behavior.

### Control variables

The relationship between maternal unpredictable work schedules and child well‐being could also be confounded by other maternal and household attributes. We adjust our models for mothers' hourly wage, usual weekly work hours, household income, job tenure, and managerial position as well as their age, race/ethnicity, school enrollment, marital status, and the age and gender of the focal child. We additionally include measures that categorize mothers by whether they are (a) single, (b) cohabiting with a partner who is employed, (c) cohabiting with a partner who is not employed, (d) married to a partner who is employed, or (e) married to a partner who is not employed. Finally, we include three measures that directly measure the nonstandard timing of mothers' shifts. Mothers are asked to rate the frequency (often, sometimes, or never) that they work (a) evening hours after 6 p.m., (b) overnight hours between midnight and 8 a.m., and (c) early morning hours before 8 a.m. We also include a set of year and month fixed effects to guard against seasonal and period effects.

Table 1 describes the analysis sample demographically and economically. The sample is disadvantaged, with 62% reporting household incomes of less than $35,000 per year and with a median hourly wage of $11.75. The average age of mothers in the sample is about 35 years, and the sample is 59% White, non‐Hispanic, 12% Black, non‐Hispanic, and 22% Hispanic. There is significant variation in marital status, with 30% reporting being single. The focal children are evenly divided by gender, with a mean age of 7.5 years.

**TABLE 1:** Descriptive statistics for The Shift Project sample of working mothers, weighted

**Household income**	
Less than $15,000	17%
$15–$25 K	23%
$25–$35 K	22%
$35–$50 K	18%
$50–$75 K	12%
$75–$100 K	5%
$100 K or more	3%
**Age**	
Mean	34.8
Median	34
**Race/ethnicity**	
White, non‐Hispanic	59%
Black, non‐Hispanic	12%
Hispanic	22%
Other or multiple	7%
**Educational attainment**	
Less than HS	6%
HS or GED	39%
Some college or more	55%
**School enrollment**	
Currently enrolled	8%
**Marital status/partner employment status**	
Single	30%
Cohabiting, partner employed	19%
Cohabiting, partner not employed	8%
Married, partner employed	36%
Married, partner not employed	8%
**Focal child age**	
Mean	7.5
Median	7
**Focal child gender**	
Female	47%
**Hourly wage**	
Mean	$12.3
Median	$11.75
**Usual number of work hours**	
Mean	33.6
Median	36
**Managerial status**	
Manager	27%
**Job tenure**	
Less than 1 year	18%
1 year	12%
2 years	12%
3 years	10%
4 years	8%
5 years	5%
6 years or more years	36%
**Evening shift**	
Never	26%
Sometimes	37%
Often	37%
**Overnight shift**	
Never	75%
Sometimes	16%
Often	10%
**Early morning shift**	
Never	36%
Sometimes	28%
Often	36%
***N***	**2613**

## ANALYTICAL APPROACH

We use the observed variation in maternal work schedules to estimate descriptive associations between mothers' exposure to unpredictable schedules and child behavior. We regress each outcome on the measures of maternal schedules controlling for individual characteristics, other job characteristics, and year and month fixed effects.

While these estimates are not causal, we note that by design, the sample has limited heterogeneity – everyone is an hourly retail or food service worker at large national chain employers and we control for economic and demographic characteristics. This limited heterogeneity is a key strength of the data as compared to sources like the NSLY‐97 that, because of sample size limitations, would have to rely on comparisons between low‐wage retail workers and other, higher‐SES workers to infer the effects of maternal precarious schedules on child well‐being. While sources of unobserved heterogeneity are thus limited by design, they are by no means eliminated and the model results must be understood as associations and not evidence taken as evidence of causal effects.

(1) $$Y_{i}=\beta_{0}+\beta_{1}{\text{Scheduling}}_{i}+\beta_{2}X_{i}+\beta_{3}J_{i}+\beta_{4}C_{i}+\gamma +\mu +E_{i}$$

In Equation (1), the outcome of interest, Y for individual i, is regressed on a set of demographic control variables, X, a set of job scheduling characteristics, J, and a set of child characteristics, C, all described above. The coefficients of interest are represented by β1 and summarize the relationship between work schedules and the well‐being of children in terms of the dependent variables described above. The terms γ and μ represent month and year fixed effects, which control for seasonality and secular trends.

We next estimate whether these associations are mediated by household economic insecurity, developmental parenting time, and maternal well‐being. We introduce the groups of measures that capture each of the mediating paths and assess whether the size and significance of the coefficients on the scheduling measures attenuates relative to the estimates in the baseline model. We use the Karlson–Holm–Breen (KHB) (2013) method to test if the mediation is statistically significant. While the KHB method is most frequently used to estimate mediation with nonlinear models, the method accommodates OLS models and provides a convenient means of accounting for multiple mediators, as we have in this instance.

## ROBUSTNESS

We test the robustness of these results to the introduction of state fixed effects and employer fixed effects. By including state fixed effects, we isolate the relationship between maternal work schedules and child behavior from any time‐invariant state‐level economic, policy, or cultural factors that may affect scheduling practices and child outcomes. By introducing employer fixed effects, we focus our identification on within‐employer variation in maternal scheduling practices. To the extent that workers sort into firms based on knowledge of scheduling practices, the introduction of these fixed effects reduces bias in the estimates. However, they do not address selection into more unpredictable schedules among a given company's workforce. We are conceptually focused on schedule unpredictability as opposed to flexibility from the worker's perspective. While it is unlikely that reported on‐call shifts, timing changes, and limited advance notice reflect desirable schedule flexibility, we also assess the robustness of our results to conditioning the sample on respondents who report that “Starting and finishing times are decided by my employer and I cannot change them on my own” or that “Starting and finishing times are decided by my employer but with my input,” and not that the employee can decide within limits or entirely on her own. Finally, we also test the robustness of the results to unweighted estimation.

## RESULTS

### Descriptive statistics

**FIGURE 1:**
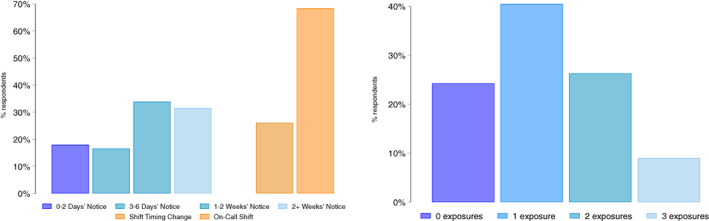
Schedule unpredictability among mothers

We describe the work schedules of mothers of focal children aged 2–15 years in Figure 1. The bars on the far left‐hand side of Figure 1 describe the amount of advance notice that mothers receive; 17% get their schedules with less than 72 hours' notice, another 13% with 3–6 days' notice, and 30% with 1–2 weeks' notice. Just 40% of mothers get more than 2 weeks' notice. The orange‐toned bars show that 25% of parents report working on call in the past month and that 65% experienced a last‐minute change to schedule timing. In the right‐hand panel of Figure 1, we show the distribution of the summed scale of maternal scheduling exposures. For mothers, one in four workers report schedule predictability—no on‐call shifts or timing changes and at least 1 week of advance notice; 40% report one of these exposures, and another 26% report two. Nearly 10% of mothers report being exposed to all three of these sources of schedule unpredictability.

## REGRESSION RESULTS

### Main estimates

We present our main regression estimates in Table 2. Each coefficient represents an estimate from a separate model that contains that focal measure of work scheduling exposure alongside the full set of control variables. In column (1), we present the estimates of the associations between maternal work scheduling and child internalizing behavior. We see that exposure to each dimension of precarious scheduling increased child internalizing—there was a significant and positive coefficient on on‐call shifts and on changes to schedule timing, though the latter was only significant at the *p* < .10 level (*p* = .056). More advanced notice was negatively related to internalizing, although the strongest association was found for those who have at least 2 weeks' advance notice of their work schedules. In column (2), we found directionally consistent associations between externalizing and on‐call shifts, although the coefficient was not significant, and we found only a marginally significant association with changes to schedule timing (*p* = .063) We found evidence of significant protective effects of advance notice of 1–2 weeks and more than 2 weeks on externalizing behavior.

**TABLE 2:** Maternal schedule unpredictability and child behavior

	(1)		(2)	
	Internalizing		Externalizing	
	β	SE β	β	SE β
*On‐call shift*				
No	(ref)	(ref)	(ref)	(ref)
Yes	0.48*	(0.23)	0.36	(0.34)
R^2^	0.113		0.122	
*Change to schedule timing*				
No	(ref)	(ref)	(ref)	(ref)
Yes	0.35+	(0.18)	0.49+	(0.26)
R^2^	0.111		0.124	
*Advance notice*				
0–2 days	(ref)	(ref)	(ref)	(ref)
3–6 days	−0.40+	(0.22)	−0.37	(0.33)
1–2 weeks	−0.29	(0.23)	0.70*	(0.32)
2+ weeks	−0.52*	(0.23)	−0.85*	(0.33)
R^2^	0.112		0.127	
Observations	2613		2613	

*** *p* < .001. **p < .01. *p < .05. +*p* < .10.

**FIGURE 2:**
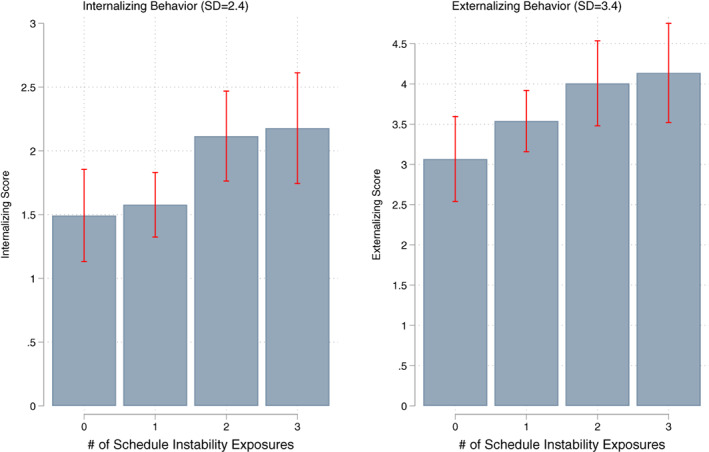
Maternal schedule unpredictability and child behavior. Predicted values with 95% CI from regression models with controls for child and mothers' demographic and work characteristics, and year and month fixed effects on weighted and multiply imputed data

Table 3 presents our estimates of the association between the maternal schedule unpredictability scale and each of the child behavior outcome measures. For internalizing, we see that compared to children whose mothers are unexposed to unpredictability, children whose mothers experience 2 or 3 exposures exhibited significantly more internalizing behavior, with the association approximately twice as large for the 9% of children whose mothers are most exposed to unpredictable scheduling. Figure 2 plots predicted values from this model to help size these associations. Moving from 0 exposures to 3 exposures was associated with a one‐quarter of a *standard* deviation increase in internalizing.

**TABLE 3:** Maternal schedule unpredictability scale and child behavior

	(1)		(2)	
	Internalizing		Externalizing	
	β	SE β	β	SE β
*Unpredictability scale*				
No sources of unpredictability	(ref)	(ref)	(ref)	(ref)
1. Source	0.08	(0.23)	0.47	(0.32)
2. Sources	0.62*	(0.26)	0.94*	(0.40)
3. Sources	0.69*	(0.29)	1.07*	(0.44)
*Evening shifts*				
Often	−0.01	(0.23)	−0.42	(0.34)
Sometimes	−0.09	(0.23)	−0.20	(0.35)
Never	(ref)	(ref)	(ref)	(ref)
*Overnight shifts*				
Often	0.37	(0.36)	0.47	(0.58)
Sometimes	0.38	(0.23)	0.11	(0.31)
Never	(ref)	(ref)	(ref)	(ref)
*Early morning shifts*				
Often	0.28	(0.21)	0.09	(0.30)
Sometimes	0.30	(0.19)	0.44	(0.28)
Never	(ref)	(ref)	(ref)	(ref)
Observations	2613		2613	
*R* ^*2*^	0.126		0.131	

*** *p* < .001. *p < .01. *p < .05. + *p* < .10.

Table 3 also presents the estimates of the association between maternal nonstandard schedule timing and child behavior. None of the measures were significantly associated with internalizing or externalizing, but the pattern of coefficients is interesting. For internalizing and for externalizing behavior, the coefficients on working overnight or early morning shifts sometimes or often were positive. Surprisingly, the coefficients on evening work were negative, though fall far short of statistical significance.

## ROBUSTNESS

**FIGURE 3:**
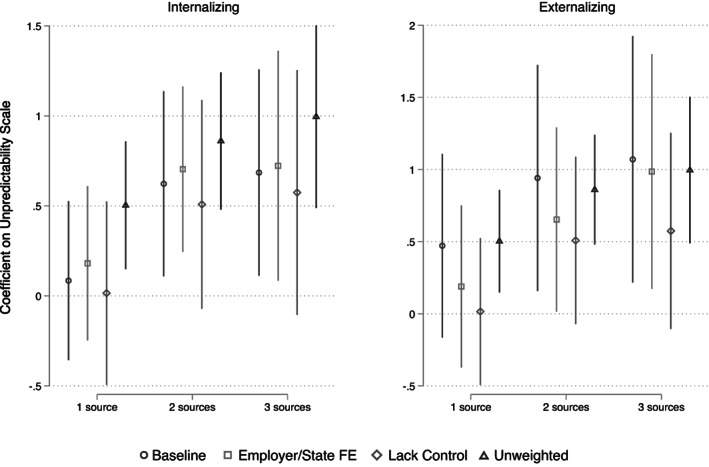
Maternal schedule unpredictability and child behavior—robustness. Predicted values with 95% CI from regression models with controls for child and mothers' demographic and work characteristics, and year and month fixed effects on weighted and multiply imputed data

Figure 3 presents plotted coefficients from Table 2 (O markers) as well as coefficients from two alternative models designed to test the robustness of these results. The first robustness test introduced state and employer fixed effects. These coefficients (□ markers) closely followed the pattern of our preferred estimates. Accounting for both time‐invariant features of states (such as aspects of the policy environment, labor market, and local cultures) and features of employers (such as differential selection into employment) did not materially affect the estimated associations between mothers' unpredictable work scheduling and child outcomes.

The second robustness test assessed if the estimates were consistent after limiting the sample to only those mothers who do not have primary control over their work schedules in order to ensure that we are observing the “instability” that is of primary theoretical interest rather than “flexibility.” These estimates (◊ markers) were generally also quite close to our preferred estimates, though in the case of 2 or 3 exposures predicting internalizing, were slightly smaller. Finally, the fourth estimates (▵ markers) were unweighted and were substantially larger than any of the weighted estimates.

## MEDIATION

**FIGURE 4:**
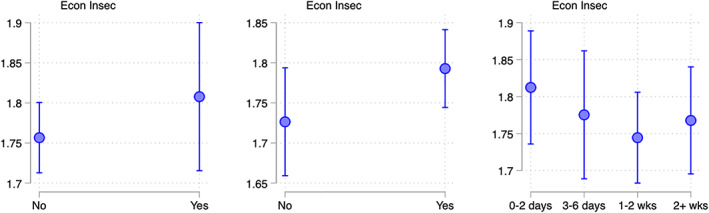
Maternal schedule unpredictability and mediating factors. Predicted values with 95% CI from regression models with controls for child and mothers' demographic and work characteristics, and year and month fixed effects on weighted and multiply imputed data

Finally, we examined whether these associations between unpredictable maternal work schedules and child well‐being were mediated by our hypothesized pathways of (1) economic insecurity, (2) lack of developmental time with children, and/or (3) diminished maternal well‐being. First, in Figure 4, we plotted predicted values for each of three mediating variables (y‐axes) by the levels of schedule instability (x‐axis). These are estimates from a version of our main regression model that included controls for child and mothers' demographics and work characteristics, and year and month fixed effects. We see that for each mediator, there were significant positive associations with work schedule instability, with association sizes on the order of a third to half of a standard deviation across the range of the scheduling exposures.

The results of our mediation analysis are summarized in Table 4. For each outcome, we show the unmediated association with our scale measure of exposure to schedule unpredictability. Each of the coefficients is relative to the base category of no exposures and is shown labeled “Total Association.” These coefficients are the same as those in Table 3.

**TABLE 4:** Mediation of maternal schedule unpredictability and child behavior

	Internalizing	Externalizing
*1. Exposure*		
Total association	0.08*	0.47
Direct association	−0.07	0.26
Indirect association	0.15	0.21
Economic insecurity	NA	NA
Time with children	NA	NA
Maternal well‐being	NA	NA
*2. Exposures*		
Total association	0.62*	0.94*
Direct association	0.43	0.68+
Indirect association	0.19*	0.26+
Economic insecurity	0%	2%
Time with children	3%	6%
Maternal well‐being	27%	19%
*3. Exposures*		
Total association	0.69*	1.07*
Direct association	0.45	0.66
Indirect association	0.24*	0.41**
Economic insecurity	1%	7%
Time with children	6%	11%
Maternal well‐being	28%	20%
Observations	2613	2613

*** *p* < .001. **p < .01. *p < .05. +*p* < .10.

We next decompose this “Total Association” for each level of unpredictability (1, 2, and 3 vs. 0) into a portion that was accounted for by our mediating variables (“Indirect Association”) and into the remaining unmediated association with unpredictability (“Direct Association”). The significance of the “Total Association,” the remaining “Direct Association,” and the “Indirect Association” is noted with asterisks. Finally, for each level of unpredictability, we show the percent of the “Total Association” that are accounted for by each of the three mediating pathways. These percentages do not sum to 100% because we are unable to completely mediate the association.

For internalizing (M1), there was no significant association between 1 exposure and internalizing behavior (and we note that these estimates were small and the percent mediated not especially meaningful given that lack of a significant direct association). The association between two exposures and internalizing (relative to no exposures) was statistically significant, and economic insecurity, time with children, and maternal well‐being were together statistically significant mediators of this relationship. We were similarly able to partially mediate the association between 3 exposures and internalizing behavior (relative to no exposure), with the three mediators together accounting for about 35% of the association. Maternal well‐being mediated the largest percentage of this relationship (27%–28%).

For externalizing (M2), we again see no statistically significant association between 1 exposure to unpredictability and externalizing behavior. For two exposures, there was a significant association with externalizing, although the mediation results were ambiguous, suggesting significant mediation, but only at the *p* < .10 level and together accounting for 27% of the association. The statistically significant association between 3 exposures and externalizing was significantly mediated by economic insecurity, time with children, and maternal well‐being, with these three factors accounting for 38% of the association. As before, maternal well‐being mediated the largest percentage of this relationships (20%).

## DISCUSSION

Service sector workers have been lionized in the recent popular imagination—recognized as “essential workers” and “heroes” who have taken substantial risk during COVID‐19 working at grocery stores, restaurants, and hardware stores, and in delivery and fulfillment—the new front lines in an epidemic where the safest place is at home. Yet these workers face extremely precarious working conditions—low wages, few fringe benefits, and unpredictable work schedules. While some prominent employers have offered temporary measures such as hazard pay, service sector jobs continue to be characterized by poor job quality.

In this article, we focus on an understudied aspect of job quality—unpredictable work schedules—and ask how mothers' exposure to such scheduling practices matters for child well‐being. We use novel data from The Shift Project to demonstrate that mothers' schedule unpredictability is associated with children's internalizing and externalizing behavior problems. Previous research has examined how nonstandard work schedules affect child well‐being, but a lack of data has precluded an examination of how unstable schedules are associated with child behavior. Therefore, these findings on schedule instability and unpredictability are a novel extension to the research literature on parental work and child well‐being.

Work schedule unpredictability takes many forms including on‐call work, shifts that are scheduled with little advance notice, and last‐minute changes to shift timing. We find that children whose parents have the most unpredictable schedules have the worst behavioral outcomes, those whose parents have the most stable schedules fare the best, and those whose parents experience moderately unpredictable schedules generally occupy an intermediate position.

The Shift Data allows us to examine potential pathways through which mothers' unpredictable work schedules may affect child well‐being. Unpredictable work schedules are associated with greater economic insecurity, less parental time spent engaging in activities with children, and diminished maternal well‐being, and the relationship between mothers' work schedules and child well‐being is mediated, in part, by each of these mediating conditions, although this mediation is only significant for parents who are exposed to the most schedule unpredictability. Maternal well‐being is a particularly strong mediator, but economic insecurity and parental time also play a role as intervening mechanisms. All of our mediators together leave a portion of the relationship between schedule unpredictability and children's behavior unexplained. Prior research establishing the relationship between unpredictable work schedules and unstable child care arrangements (Henly & Lambert, 2005), which was not measured in our analysis but represents another potential mediating pathway.

These findings point towards the intergenerational consequences of low‐wage work. Prior research and policymaking have often focused on economic deprivation as a key driver of intergenerational disadvantages. Complementing and extending this narrative, we show that the temporal dimensions of low‐wage work that influence parents' time and stress as well as their economic security have important implications for child development. When parents experience routine instability in their work schedules, we see downstream consequences for children's internalizing and externalizing behaviors. This temporal disadvantage compounds economic disadvantages faced by workers in the service sector and their children.

Currently, a handful of cities and states including San Francisco, Seattle, Emeryville in California, New York City, Oregon State, Philadelphia, and Chicago have passed local legislation to encourage more stable work schedules in the service sector and several other cities and states are considering similar legislation. Our research suggests that these new labor regulations, if successful in creating more stable work schedules for parents, could have important collateral benefits for children.

The Shift Data allows us to examine relationships that have previously been suspected but not systematically researched because of data limitations. However, several limitations of the Shift Data should be kept in mind. The relationships examined in this article are based on the parental self‐reports of work schedules, mediators, and child outcomes. Therefore, these data are subject to shared method bias that can occur when a single informant reports on predictors and outcomes. In this article, shared method bias would occur if the same child behavior was perceived as more problematic by parents with unstable schedules than by parents with stable schedules. This could be the case, for instance, if the stress of uncertain schedules led parents to have less patience and tolerance for children's acting out and to view children's externalizing behavior in a more negative light. It is worth noting that this limitation is not unique to the Shift data. Many datasets, such as the Fragile Families and Child Wellbeing surveys, also face this same source of potential bias.

Although capturing a novel set of unpredictable scheduling practices is a unique contribution of The Shift Project survey, the survey does not capture the frequency of unpredictable schedule practices. A valuable direction for future research would be to more fully capture the frequency and intensity of exposure to schedule unpredictability and its association with child outcomes.

The Shift Project data allow unprecedented insight into how uncertainty in parental work schedules is associated with child behavior, but the data come from a non‐probability sample. Comparisons of the Shift data with national longitudinal survey (NLS) and CPS data sources have found the Shift data to be in line with these probability samples (Schneider & Harknett, 2019b), and the analyses presented here are weighted to the characteristics of parents in the same industries and occupations surveyed by the American Community Survey. But, the sample of mothers in Shift may not be fully representative of the population of mothers working in the service sector. The Shift data cover large employers and do not represent workers employed by smaller, non‐chain employers. The implications of schedule unpredictability for children could differ between large and small employers if, for instance, employer size is correlated with the frequency of exposure to unpredictable scheduling. Therefore, there may be limitations to the generalizability of the results in particular to workers employed at smaller‐sized employers.

More generally, these findings pertain to service sector workers, which is both a strength and a limitation. The strength of our focus is that the service sector represents a strategic site, given the prevalence of schedule instability and the targeting of scheduling legislation to this sector. This sector also employs nearly one in five U.S. workers. The limitation of our focus is that the relationships between schedule and unpredictability and child behavior could differ in other occupational sectors where pay is higher and schedule control is greater. In fact, these types of work characteristics may explain why an earlier study—of a sample of mothers in the Fragile Families and Child Wellbeing study who were employed in a broad set of industries—did not find a relationship between working different times each week and child behavior (Dunifon et al., 2013).

A final limitation involves ambiguity in time ordering of relationships. Our data come from repeat cross sections; therefore, we cannot be certain about the causal ordering among our predictors, mediators, and outcomes. One area of particular concern is that parental well‐being may, in part, causally influence work schedule instability if parents with lower levels of well‐being are less reliable workers and are penalized with worse schedules. Another possibility is that children's externalizing behavior could interfere with parents' ability to fulfill work obligations, perhaps by making it difficult to arrange child‐care. Therefore, when interpreting our results, we must keep in mind that the possibility of reverse causality for some of our measured relationships could lead us to somewhat overestimate the influence of schedules on mediators and child outcomes. The Shift Project is collecting longitudinal data from a subsample of workers, which in the future will offer some leverage to reduce ambiguity in the time ordering of these relationships.

Using the newly available data from The Shift Project, we have presented evidence of a striking relationship between the instability and unpredictability of parental work schedules and children's behavioral problems. Service sector jobs are sometimes characterized as temporary jobs for teenagers, but a sizeable share of service sector workers are parents whose children are affected by the temporal instability introduced by the demands of these jobs. For workers with higher socioeconomic status, demanding jobs are often accompanied by generous compensation packages. But, in the low‐wage service sector, jobs are frequently demanding, often requiring workers to be available 24/7 and on short notice, but financial compensation and fringe benefits are meager. In this context, the portrait that emerges is one of the children facing an accumulation of disadvantages and barriers to intergenerational mobility: Economic resources are low, parental time is constrained, and maternal well‐being is reduced.
